# Unnecessary Use of Corticosteroids for managing early mild symptoms of COVID-19 may lead to Rhino-ortibal-cerebral mucormycosis in Patients with Diabetes – a case series from Lahore, Pakistan

**DOI:** 10.1177/20499361221097417

**Published:** 2022-05-06

**Authors:** Somia Iqtadar, Masooma Hashmat, Muhammad Nabeel Akbar Chaudhry, Sami Ullah Mumtaz, Sajid Abaidullah, Domingo A. Pascual-Figal, Amjad Khan

**Affiliations:** Department of Medicine, King Edward Medical University, Lahore, Pakistan; Department of Medicine, King Edward Medical University, Lahore, Pakistan; Punjab Institute of Cardiology, Lahore, Pakistan; Department of Medicine, King Edward Medical University, Lahore, Pakistan; Department of Medicine, King Edward Medical University, Lahore, Pakistan; University of Murcia, Hospital Universitario Virgen de la Arrixaca, Murcia, España; INEOS Oxford Institute for AMR Research, University of Oxford, UK; Nuffield Division of Clinical and Laboratory Sciences (NDCLS), Radcliffe Department of Medicine, John Radcliffe Hospital, University of Oxford, UK

**Keywords:** corticosteroids, COVID-19, diabetes mellitus, invasive fungal infection, rhino-orbital-cerebral mucormycosis

## Abstract

Rhino-orbital-cerebral mucormycosis (ROCM), a rare but fatal fungal infection, has recently emerged as a serious complication after corticosteroids therapy in COVID-19 patients, predominantly in diabetic and immunocompromised patients. The World Health Organization (WHO) COVID-19 current guidelines recommend corticosteroids administration in hospitalized COVID-19 patients requiring supplementary oxygen or mechanical ventilation. Herein, we report a case series of seven patients with COVID-19; three mild, three moderate, and one severe, from Lahore, Pakistan; all were using corticosteroids for managing their early mild symptoms of COVID-19 at home for around 2–3 weeks without a physician’s advise, presented, and admitted with ROCM to Mayo hospital, Lahore, from March to June 2021. Out of the seven patients, five patients had uncontrolled diabetes mellitus (DM) as comorbidity. Eye pain, facial swelling and pain, nasal blockage, and black coloration around eyes, on palate, and oral mucosa were the presenting complaints at the time of admission. All the patients had radiographic imaging, including computed tomography (CT), paranasal sinuses (PNS), or brain magnetic resonance imaging (MRI) carried out at the hospital, which confirmed mucosal thickening and adjacent sinus bony erosions with intracranial extension. All the patients were treated with local debridement of the infected necrotic tissue along with intravenous liposomal Amphotericin B and Posaconazole or Amphotericin B depending on the case. Due to timely management, in six out of seven patients, prognosis was good due to early diagnosis and treatment, while one patient with severe COVID-19 illness deteriorated and died. The misuse of corticosteroids for managing early mild symptoms of COVID-19 in diabetic and other immunocompromised patients can lead to fatal ROCM, which can further increase their risk of developing severe COVID-19 and mortality. It is stressed that only physician’s recommended therapeutic advice should be followed for managing early mild symptoms of COVID-19 in self-isolation and avoid the unnecessary use of corticosteroids. This case series also emphasizes that COVID-19 diabetic patients treated with corticosteroids need more vigilant monitoring and high suspicion of early diagnosis and treatment of invasive fungal infection. Early diagnosis and management can reduce morbidity and mortality.

## Introduction

The sustained prevalence of the COVID-19 pandemic and the efforts to mitigate the spread have continued to disrupt much of the world population. There is currently no specific and conclusively proved effective treatment available for COVID-19. Corticosteroids, due to its anti-inflammatory mechanism, are currently the most widely used drugs for the treatment of hospitalized patients with COVID-19 to prevent mortality. The RECOVERY trial reported the beneficial effects of dexamethasone in reducing mortality in COVID-19-infected patients requiring supplemental oxygen and mechanical ventilation;^1^ however, no survival benefit have been observed in patients who do not require supplemental oxygen during the course of illness. On 2 September 2020, the World Health Organization (WHO) recommended the treatment of corticosteroids in severe and critically ill COVID-19 patients under medical supervision.^2^

In the midst of this COVID-19 pandemic, there has been a recent sudden surge in the global incidence of rhino-orbital-cerebral mucormycosis (ROCM) and aspergillosis in patients with COVID-19 after treatment with corticosteroids, and uncontrolled diabetes mellitus (DM) was found to be a common comorbidity amongst the majority of these patients.^3–14^ As of 7 June 2021, India has reported the highest 24,370 cases of mucormycosis in active and recovered COVID-19 patients, including 17,601 cases with a history of DM.^6^ The development of aggressive ROCM fungal infection in diabetic patients suggests a possible clinical relationship between SARS-CoV-2, corticosteroid administration, and uncontrolled DM. The potential and fatal risk of mucormycosis has now emerged as another serious complication that must be carefully considered, especially, when immunocompromised and DM COVID-19 patients are treated with corticosteroids.

**Figure 1.**
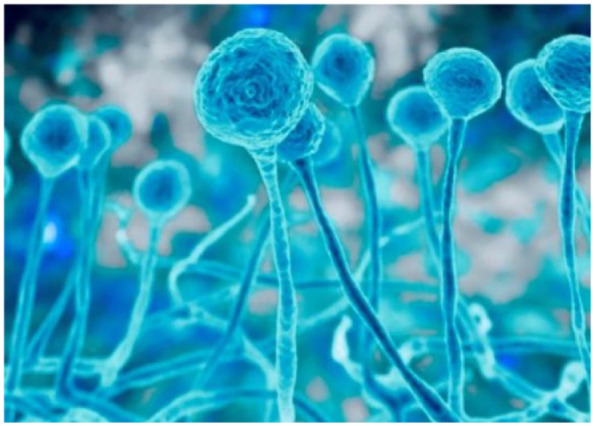
Colony of the Mucor fungi (Mucorales), also known as black fungus, causes mucormycosis (or black fungus disease).

Mucormycosis, commonly known as black fungus disease, is a rare, time-sensitive, opportunistic but devastating invasive fungal infection (IFI) caused by the order Mucorales (Figure 1),^15,16^ and associated with a high case fatality rate of 46%,^17^ attributed in part to their intrinsic high level of resistance to multiple antifungal drugs.^18,19^ Early in the course of infection, diagnosis can be almost impossible. In the spectrum of COVID-19 disease, the most common type is the ROCM reported to date, mostly in patients with uncontrolled DM or corticosteroid exposure.^11^ The symptoms of ROCM include one-sided facial swelling, headache, fever, nasal or sinus congestion, black lesions on nasal bridge or upper inside of mouth, double or blurred vision, and partial or complete loss of vision in one or both eyes, which rapidly becomes more severe. ROCM occurs due to inhalation of spores in the paranasal sinuses of at-risk hosts.^20^ Tissue necrosis is a hallmark of mucormycosis, resulting from vascular invasion and thrombosis.^21^ Susceptible persons for mucormycosis include people with diabetes with poor glycemic control, those on systemic corticosteroid, and immunocompromised patients.^22^ DM, particularly with ketoacidosis, is the commonest reported risk factor in non-COVID-19 mucormycosis, predominantly in middle- and low-income countries.^23–25^ Mucormycosis treatment entails the use of costly and toxic antifungal agents like Amphotericin B and Posaconazole which must be administered for a prolonged period. The need of risky and potentially disfiguring surgical interventions adds significantly to the mortality and morbidity of this disease.

In Pakistan, recently, there has also been a notable rise in incidence of IFI in patients with COVID-19 treated with corticosteroids, and most of these patients had DM as the common comorbidity. Nasir *et al.*^26^ first reported the cases of COVID-19-associated pulmonary aspergillosis (CAPA) in COVID-19 patients with DM as the predominant comorbidity (4/5). This first reported study showed an overall fatality rate of 44% (4/9) from CAPA. In the present study, we report a case series of seven patients with COVID-19, mostly diabetic, who developed ROCM after unnecessarily using corticosteroids for managing their early mild symptoms of COVID-19 at home. The aim of the present study is to emphasize that the misuse use of corticosteroids for managing early mild symptoms of COVID-19 infection in self-isolation may lead to the development of fatal IFI of mucormycosis especially in diabetic and immunocompromised patients. Given the poor prognosis of this disease and its time liability, these patients group should be closely monitored for sequelae of immunosuppression during and after COVID-19 treatment with corticosteroids.

## Case series

**Figure 2.**
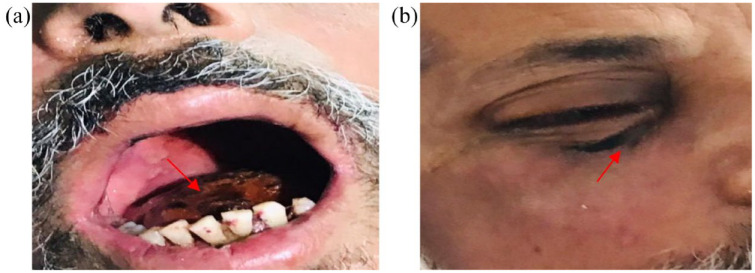
Symptoms of mucormycosis or black fungus disease reported in COVID-19 patients after misuse of corticosteroids: (a) black coloration of oral cavity mucosa and (b) black coloration around right eye.

**Figure 3.**
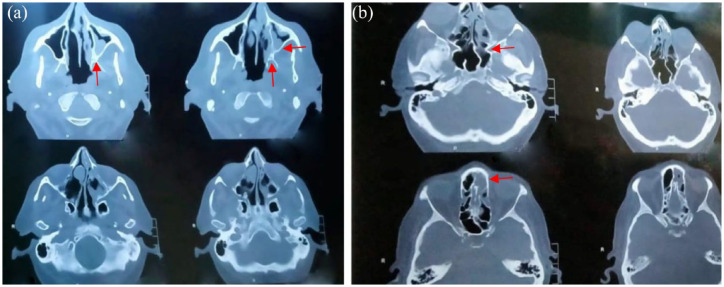
Contrast-enhanced computed tomography (CECT) images showing involvement of (a) maxillary and ethmoidal sinuses and (b) maxillary sinuses.

A summary of this case series involving seven patients is presented in Table 1. The mean age of the patients was 53 ± 10 years, with five patients had uncontrolled DM as comorbidity. All the patients had confirmed COVID-19 infection (shown by reverse transcriptase polymerase chain reaction-based positive nasopharyngeal swab) and typical symptoms of ROCM at the time of admission. All the patients had used corticosteroids for management of their early mild symptoms of COVID-19 at home for around 2–3 weeks and subsequently developed biopsy-proven mucormycosis (Figure 2) including two patients with aspergillosis. All the patients had radiographic imaging including, computed tomography (CT), paranasal sinuses (PNS), or magnetic resonance imaging (MRI) of the brain carried out at the hospital, which confirmed mucosal thickening and adjacent sinus bony erosions with intracranial extension (Figure 3). At our hospital, all these patients were treated with local debridement of the infected necrotic tissue along with intravenous liposomal Amphotericin B and Posaconazole or Amphotericin B depending on the case. Amphotericin B was started at 5 mg/kg per day and exceeded up to 10 mg/kg per day in slow infusion (depending on extent of diseases and tolerability) with careful monitoring of side effects and serial measurement of renal function. In case of nephrotoxicity, dose was either reduced or switched to Posaconazole. Dose of Posaconazole used was 600 mg/day in two divided doses as loading dose on day one followed by 300 mg once a day as maintenance therapy. Antifungal drugs were given pre-surgery and post-surgery for a period of 3–6 months. Overall, in six out of seven patients, prognosis was good due to early diagnosis and treatment, while one patient with severe COVID-19 illness deteriorated and died, possibly due to combination of sepsis, end-stage renal disease (ESRD), and superimposed secondary IFI.

**Table 1.** Demographic and clinical details of a case series of seven COVID-19 patients who developed invasive fungal infection after unnecessarily using corticosteroids for managing early mild symptoms of COVID-19 at home.

Patient’s ID	1	2	3	4	5	6	7
Age (years)	44	65	68	48	54	55	38
Sex	Female	Female	Male	Female	Male	Male	Male
Comorbidities	DM	DM, HTN, IHD	DM, HTN, CKD	HTN, ESRD	DM	DM	Asthma
COVID-19 Severity	Mild	Moderate	Moderate	Severe	Mild	Mild	Moderate
Used steroids for COVID-19	Yes	Yes	Yes	Yes	Yes	Yes	Yes
Supplementary O_2_ requirement at admission	No	No	No	Yes	No	No	No
Presenting symptoms of mucormycosis	Headache, facial, eye pain, and swelling	Facial, eye pain, swelling, nasal blockage, loss of vision in left eye, black coloration around eyes, palate, and oral mucosa	Painful loss of vision in left eye, facial pain, ptosis, black coloration around eyes, palate, and oral mucosa	Facial pain and swelling, black coloration around eyes, palate, and oral mucosa	Facial pain and ptosis, black coloration around eyes, palate, and oral mucosa	Fever, eye facial pain, and swelling, black coloration around eyes, palate, and oral mucosa	Facial pain and nasal blockage
HPE and fungal smear	Yes Mucormycosis + Aspergillosis	Yes Mucormycosis	Yes Mucormycosis	Yes Mucormycosis	Yes Mucormycosis + Aspergillosis	Yes Mucormycosis	Yes Mucormycosis
Radiological evidence	Yes	Yes	Yes	Yes	Yes	Yes	Yes
Treatment	Local debridement (lateral rhinotomy), Amphotericin and Posaconazole	Local debridement and Amphotericin + Posaconazole	Local debridement, FESS and Amphotericin + Posaconazole	Local debridement and Amphotericin	Local debridement, FESS + Septoplasty and Amphotericin	Local debridement and Amphotericin	Local debridement and Amphotericin
Clinical outcome	Improved	Improved	Improved	Died	Improved	Improved	Improved

CKD, chronic kidney disease; DM, diabetes mellitus; ESRD, end-stage renal disease; FESS, functional endoscopic sinus surgery; HPE, histopathology examination; HTN, hypertension; IHD, ischemic heart disease.

## Discussion

Currently, corticosteroids are considered as essential therapy in hospitalized COVID-19 patients requiring supplemental oxygen, because of its anti-inflammatory, antioxidant, pulmonary vasodilator, and anti-edematous pharmacological mechanism. Corticosteroid administration in patients with severe acute respiratory distress syndrome (ARDS) has been previously reported to reduce mortality and the duration of intermittent mandatory ventilation.^27–30^ However, its pros and cons in ARDS still remains controversial. Corticosteroids are immune-suppressive agents and have also associated severe adverse effects such as delayed viral clearance, poor glycemic control, and secondary opportunistic infection.^31–33^ Patients with COVID-19 are administered with high doses of corticosteroids resulting in weakening of the immune system, hence making the host susceptible to the development of secondary infections such as mucormycosis. In addition, corticosteroids treatment can lead to elevated blood sugar level, which is challenging for patients with uncontrolled DM and the acidic environment due to this condition favors the fungal (Mucorales) growth. Inhalation of filamentous fungi by patients also weakens their immune defense pathways. COVID-19-associated mucormycosis (CAM) has been mostly reported in patients with a history of DM, suggesting that patients with DM are more susceptible to COVID-19 infection associated with mucormycosis infection.^5,11,34–39^

The current WHO guidelines recommend corticosteroids administration strictly for the treatment of severe and critically-ill COVID-19 patients, but not for patients with non-severe COVID-19 symptoms. The unregulated and improper use of corticosteroids (i.e. dexamethasone, hydrocortisone, or prednisone) for treating even mild symptoms of COVID-19 is believed to be the primary reason for the highest cases of mucormycosis infection reported during the second COVID-19 wave in India.^5,11,34–39^ Most of these cases were in patients with a history of DM.

In patients of COVID-19 infection treated with corticosteroids, there has also been increasing reports of fatal aspergillosis.^40–42^ One case series reported an incidence of 19.4% COVID-19 associated with aspergillosis in mechanically ventilated patients.^43^ White *et al.*^44^ in a multicentre study emphasized the need for increased testing for IFI in patients with severe COVID-19 admitted in intensive care units. They reported 14.1% cases of superimposed CAPA and independent risk factors were found to be the use of corticosteroids and chronic respiratory disease.

The underlying immunological mechanism that stimulate the development of fungal infections in patients with COVID-19 still remains unknown; although several mechanisms have been proposed for CAM. It is speculated that SARS-CoV-2 affects the pancreatic β-cells leading to impaired insulin secretion thereby promoting diabetes and may also result in damage to the airway epithelium after a cytokine storm and lymphocyte depletion compromising the immune response.^45,46^ In another proposed mechanism, glucose-regulated protein 78 (GRP78) is believed to act as a co-receptor for the receptor-binding domain (RBD) of SARS-CoV-2 and allow the entry of the virus into the cells.^47^ After entering the cells, SARS-CoV-2 proteins possibly trigger unfolded protein response at endoplasmic reticulum (ER) resulting in higher expression and levels of GRP78 as reported in COVID-19 pneumonia patients.^48^

In treatment of patients with COVID-19 and DM, during corticosteroids administration blood glucose levels should be continuously monitored to maintain glycemic control and corticosteroid dosages be carefully adapted for consideration of the intended benefits. Following corticosteroids treatment, COVID-19 recovered patients with DM and other risk factors should be educated about the sign and symptoms of mucormycosis and be advised to seek urgent medical treatment should they suspect any sign of this deadly fungal infection. The identification of patients at risk, strict glycemic control, and avoidance of unnecessary use of corticosteroid in non-severe COVID-19 symptoms can help in preventing the complication of fatal fungal infection.

Mucormycosis is a life-threatening rare infection encountered in diabetic and immunocompromised individuals. Timely diagnosis is paramount in cases of mucormycosis. Patients with suspected or confirmed mucormycosis should be treated as a medical emergency; they should be referred to institutions that can perform highest level of care. The interprofessional approach to ROCM involving infectious disease and medical intensivists, otolaryngologists, physicians, ophthalmologists, radiologists, histopathologists, neurosurgeons, pharmacists, and neurologists can escalate the diagnosis and treatment and reduce mortality and morbidity. Despite these measures, the result remains poor. So a high degree of suspicion is made in high-risk individuals to accomplish a multifaceted approach.

## Conclusion

In conclusion, early-stage mild symptomatic COVID-19 patients in community, particularly those with a history of DM and other risk factors, should avoid the unnecessary use of corticosteroids as it may lead to suppression/weakening of their immune system making them more susceptible to the development of secondary infection such as ROCM and progression to severe illness and mortality. It is emphasized that community-based COVID-19 patients should follow the therapeutic advice of their general practitioner for management of their symptoms. Moreover, the primary healthcare systems should raise awareness on COVID-19 symptoms management. In the middle- and low-income countries, the misuse of corticosteroids should be strongly discouraged, and strict regulatory guidelines and measures should be placed to allow provision of corticosteroids only on physician’s prescription. Given the widespread use of corticosteroids in hospitalized severe to critically ill patients with COVID-19, physicians should be alert and be aware of the possibility of secondary IFI especially in patients with DM and other high-risk factors. A good control of glucose level preventing hyperglycemia minimizes the chances of opportunistic infections and improves prognosis. Careful assessment of all patients who are suffering from COVID-19 to classify them into mild, moderate, and severe disease and adherence to guidelines of treatment increase the chances of cure without complications. Special emphasis should be given on early recognition of super added infections in COVID-19, particularly fungal infection. When the presence of necrotic tissues and systemic sign of COVID-19 infection become manifest, especially in immunocompromised patients, mucormycosis must be considered. Early diagnosis and management can reduce morbidity and mortality.
